# Digital health solution for monitoring and surveillance of Amyotrophic Lateral Sclerosis in Brazil

**DOI:** 10.3389/fpubh.2023.1209633

**Published:** 2023-08-25

**Authors:** Ingridy M. P. Barbalho, Aleika L. A. Fonseca, Felipe Fernandes, Jorge Henriques, Paulo Gil, Danilo Nagem, Raquel Lindquist, Thaisa Lima, João Paulo Queiroz dos Santos, Jailton Paiva, Antonio H. F. Morais, Mário E. T. Dourado Júnior, Ricardo A. M. Valentim

**Affiliations:** ^1^Laboratory of Technological Innovation in Health (LAIS), Federal University of Rio Grande do Norte (UFRN), Natal, Brazil; ^2^Department of Informatics Engineering, Center for Informatics and Systems of the University of Coimbra, Universidade de Coimbra, Coimbra, Portugal; ^3^Department of Physical Therapy, Rio Grande do Norte Federal University, Natal, Brazil; ^4^Brazilian Ministry of Health, Brasília, Brazil; ^5^Advanced Nucleus of Technological Innovation (NAVI), Federal Institute of Education Science and Technology, Natal, Brazil; ^6^Department of Integrated Medicine, Federal University of Rio Grande do Norte (UFRN), Natal, Brazil

**Keywords:** public health, rare diseases, public policy, health indicators, health information system, epidemiological monitoring

## Abstract

Amyotrophic Lateral Sclerosis (ALS) is a complex and rare neurodegenerative disease given its heterogeneity. Despite being known for many years, few countries have accurate information about the characteristics of people diagnosed with ALS, such as data regarding diagnosis and clinical features of the disease. In Brazil, the lack of information about ALS limits data for the research progress and public policy development that benefits people affected by this health condition. In this context, this article aims to show a digital health solution development and application for research, intervention, and strengthening of the response to ALS in the Brazilian Health System. The proposed solution is composed of two platforms: the Brazilian National ALS Registry, responsible for the data collection in a structured way from ALS patients all over Brazil; and the Brazilian National ALS Observatory, responsible for processing the data collected in the National Registry and for providing a monitoring room with indicators on people diagnosed with ALS in Brazil. The development of this solution was supported by the Brazilian Ministry of Health (MoH) and was carried out by a multidisciplinary team with expertise in ALS. This solution represents a tool with great potential for strengthening public policies and stands out for being the only public database on the disease, besides containing innovations that allow data collection by health professionals and/or patients. By using both platforms, it is believed that it will be possible to understand the demographic and epidemiological data of ALS in Brazil, since the data will be able to be analyzed by care teams and also by public health managers, both in the individual and collective monitoring of people living with ALS in Brazil.

## 1. Introduction

Amyotrophic Lateral Sclerosis (ALS) is a rare neurodegenerative disease affecting the brain and spinal cord's upper and lower motor neurons (1). The main characteristic of this disease consists of the progression of muscle paralysis in an irreversible manner, which reflects in motor neuron degeneration causing the patient's death due to respiratory blockage (2, 3). With its etiology still unknown, the diagnosis of ALS becomes complex and, in some cases, time-consuming (4, 5). ALS has no cure yet, there are only some palliative care and treatments that can slow the progression of the disease and increase patient lifespan (6, 7). It is estimated that the life expectancy of patients ranges from 2 to 5 years after the onset of symptoms (8), but there are reported cases that far exceed this period, such as that of the famous theoretical physicist Stephen William Hawking.

In terms of epidemiology, Longinetti and Fang (9) have stated that the incidence of ALS worldwide is between 0.6 and 3.8 per 100,000 people per year. The prevalence, meanwhile, is between 4.1 and 8.4 per 100,000 people. Although a determining factor for the disease cause has not yet been found, projections show that ALS cases worldwide will escalate from 222,801 in 2015 to 376,674 in 2040, representing a 69% increase (10). Population studies show a heterogeneous distribution of ALS worldwide, with varying incidence and prevalence rates, being highest in the United States and Europe and lowest in Asia, Africa, and the Hispanic population (11–13).

Even though ALS was discovered many years ago, data about characterize the origin, accurate diagnosis and cure for the disease remains undefined. Many researchers underscore studies that clearly and specifically provide information that can help in the development of effective ALS diagnosis and treatment strategies (14, 15). The lack of clinical and epidemiological studies hinders the obtention of reliable data on the etiology, incidence, and prevalence of ALS worldwide (16).

As in the scenario of other chronic diseases, population-based studies, such as population-based registries, have proven to be important tools for defining clinical and prognostic characteristics (17). Nowadays, it is possible to find ALS-specific electronic reference registries that can serve as a basis for the development of new registries for countries that do not yet have this tool (18–23). Of note, few countries have epidemiological data on the disease, a fact that may contribute to the slower progress of ALS-related studies (24). Furthermore, studies that have explored the epidemiology of ALS in the Latin American population are scarce.

In Brazil, some studies have been conducted to indicate the prevalence and incidence in the country. According to Lopes-Júnior et al. (25), Brazil is gaining attention when it comes to public health policy discussions for rare disease patients, but public health services still have many challenges to overcome. According to the website of the Brazilian Institute of Geography and Statistics (IBGE), Brazil has a little more than 8.51 million km^2^ of territory divided among the 27 federative units, including the Distrito Federal, the country's capital, with more than 213 million inhabitants (26). Although, according to the 1988 constitution, health is a “right of all and a duty of the State”, Brazil has significant social inequality and part of the population has difficulty accessing basic health services (27, 28). Due to these characteristics, reaching the population is one of the difficulties faced in monitoring and surveillance of several diseases. Some challenges are reflected in the case of ALS patients, where the geographic distribution of these patients is still partially known.

Even with the reported challenges, some studies have already been developed. Dietrich-Neto et al. (29) conducted the first nationwide study, estimating prevalence and incidence of 0.9–1.5/100,000 and 0.4/100,000, respectively. The survey was conducted by sending a form to 2,505 Brazilian neurologists in the period between January and September 1995. Data from 443 patients were collected. The second population-based study, conducted by Moura et al. (30), had as its information source the data from death certificates, from January 2004 to December 2013. As a result, the incidence was estimated to be 0.61 to 0.89/100,000. Other studies on the epidemiology of ALS have been conducted, but only at the state or municipal level (31–34).

Despite these studies and the official documents made available for the optimization of diagnosis and proper care for ALS patients (35), the Brazilian Health System (SUS) still experiences an absence of digital health solutions that provide ALS monitoring and surveillance in the country. Considering the scarcity of epidemiological data on ALS throughout the country, it is evident the need for the implementation of digital tools for data collection and processing to generate dashboards with indicators and information that can contribute to the more effective conduct of public health policies in Brazil. The epidemiological indicators are essential resources for the situational analysis of ALS and provide subsidies that guide the development of public policies, lines of care, and effective actions in the healthcare network.

In this context, electronic records are used for the organized and accurate collection of a set of essential data for research and surveillance (36, 37). From this data, it becomes possible to present indicators and know the profile of the disease, and the data set collected in rare disease registries serves as a basis for several studies (18, 38–41). These electronic records are seen as a digital health tool that contributes to more efficient interventions in the country's health system (42, 43). Digital health solutions are strategic and innovative tools used to address health needs with the potential to improve coverage of health services and practices (44). An intelligent and responsive digital health system has benefits, especially in low-resource settings, such as low/middle-income countries (45). The reach that technologies allow us to achieve is an important factor for the realization of a national study with territorial dimensions as extensive as those of Brazil (46).

This study aims to describe and analyze the development and application of a digital health solution for research, intervention, and strengthening the response to ALS in the SUS. The development of this solution is part of a project, called RevELA (project No: 132/2018), funded by the Brazilian Ministry of Health (MoH). The RevELA project is composed of several dimensions, which demand actions with goals focused on the development of applied research and products in the areas of alternative communication (47), health education, follow-up and monitoring of patients with ALS (24, 48–51) and development of clinical protocols for cardiorespiratory rehabilitation of patients with ASL (52, 53). The present digital health solution is a platform that integrates the Brazilian National Registry of people with ALS, a tool for collecting data on individuals, and its database feeds a second tool, a nationwide monitoring room, called the Brazilian National ALS Observatory.

The national ALS monitoring room in Brazil includes resources that offer timely, transparent, and quality information, capable of providing a basis and support for the design of the profile of this grievance at the national level. The information stored on the platform can be analyzed by care specialist teams and also by public health managers, both in the individual and collective monitoring of people living with ALS and in research and public health intervention nationwide.

## 2. Materials and methods

The process of developing the digital solution was guided by the action-research method, as defined by Tripp (54), characterized by constant improvement, analysis, study, and application of changes. It is an agile continuous improvement method. The author states that action research requires both the fields of practice and scientific research because it uses research to guide actions that improve practice, that is, research acts to solve real-world problems.

**Figure 1:**
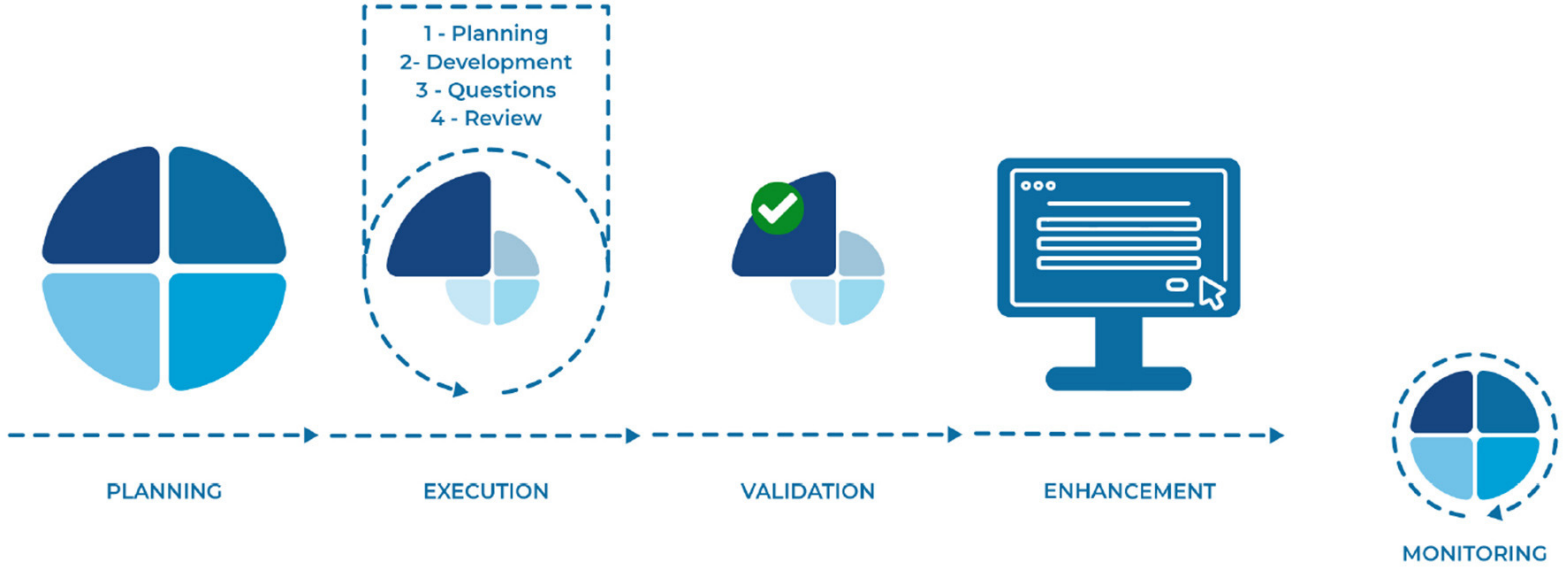
Digital solution development process.

The proposed digital health solution was developed in five stages: planning, execution, validation, implementation, and monitoring. All stages were carried out sequentially in several cycles by applying the iterative and incremental model. The cyclical nature of development composed of the defined stages led to the use of the SCRUM framework (55), shown in Figure 1. This framework is suitable for the development of the platform because it is flexible to the necessary changes and allows for continuous improvement in the development process, as well as making use of up-to-date tools, processes, and training (56).

The first stage of development consisted of planning the digital health solution. During this time, the outline, goals, technologies, architecture, functionalities, and flow of the platform were defined. After the overall planning, the solution proposed was divided into smaller functionalities to be developed iteratively and incrementally. For the development of each functionality, new planning was required to define the activities, teams, time, and effort needed. During the execution of these activities (second stage), meetings were held with the development team and the expert team to understand what impediments existed and how difficulties could be overcome so that the execution of the activities could progress. Once the execution was completed within the given timeframe, the functionality/implementation went through a validation and verification stage (third stage) with the specialists so that it could be enhanced in the final product (fourth stage). This cycle was repeated several times until the product was finished. Even after finalization, the digital health solution constantly goes through the monitoring process, in which the end user uses the platform and checks if any functionality needs to be implemented and updated.

All steps in the development of the National ALS Registry and National Observatory were monitored and validated by a transdisciplinary group of experts in the field of data science and health, with knowledge related to ALS and public health monitoring, planning, and management. The step of creating the forms for the National Registry data collection was carried out in partnership with teams of neurologists from centers considered to be reference centers for ALS in Brazil, such as: Onofre Lopes University Hospital of the Federal University of Rio Grande do Norte, University of São Paulo Medical School and Brazilian Academy of Neurology (BAN). This stage included several meetings for suggestions and validation until the definition of a minimum data set with the main variables that should be considered for collection. It is noteworthy that the National Register is not an electronic patient record, but an Epidemiological Register with national coverage in Brazil.

For the construction of the national monitoring room, besides the validation of neurologists, epidemiologists, statisticians, and members of the management team of the Public Health Secretariat of the Rio Grande do Norte (SESAP/RN) were also consulted. It is important to note that at various times during the development of the platforms, the technical team of Brazil's MoH received technical reports that allowed them to monitor and evaluate the evolution of digital health solutions. It bears mentioning that all the stakeholders involved gave their opinions and contributed in some way to the proposed analyses. This entire qualification and validation flow is a very important step in the development process since it is essential to adapt the functionalities to meet the needs of the public that will use the system and always with the focus on the final goal which is to propose an environment that allows the extraction of relevant information about ALS to assist in decision making and health interventions.

## 3. Results

### 3.1. Brazilian National ALS Registry

In Brazil, the data concerning ALS patients are still scarce. From this perspective, the Brazilian National ALS Registry emerges as an alternative to consolidating data, information, and knowledge about ALS throughout the Brazilian territory by collecting data that will help researchers and managers to better understand the disease. Within the RevELA Project, the National Registry can be understood as a structured platform, developed according to the needs defined, based on the problems already addressed, and using observational study methods that can provide subsidies for studies and research related to ALS. Its main goal is to gather data from patients diagnosed with ALS in Brazil, aiming to better understand the geographical distribution of cases and evaluate the main socio-demographic aspects. In the field of public health management, the National Registry has its architecture designed to assist in the production of knowledge that can more adequately guide the planning of health interventions, as well as support decision processes. From the patient's perspective, the platform is a reliable provider of information about the disease.

The platform development began in 2020 with the primary goal of gathering data on the incidence and prevalence of ALS in Brazil. Initially, a systematic review was performed to understand the structuring and electronic architecture of motor neuron disease registries worldwide (24). Along with this knowledge obtained from the literature, the planning and execution stages were carried out with interviews and meetings always counting on the presence of stakeholders (interested parties), as described in the methods section. From all the theoretical and practical knowledge acquired, essential requirements for the registry development were identified, highlighting the functionality to access the platform and the data collection process. For both functionalities, the platform was structured in two modules: (1) the physician's module, which allows the physician to access and register patients on the platform; and, (2) the patient module, or Self-Report, which allows the patient to access and enter their data into the platform (described in Section 3.1.1 and 3.1.2. respectively).

As a fundamental part from the point of view of information security and access to patient data, the platform adheres to and respects the guidelines described by Lei N° 13.709, called Lei Geral de Proteção dos Dados (LGPD) of Brazil (57). Its main objective is to protect the privacy and the rights of personal data subjects while respecting legal and ethical standards regarding the protection of users' data. Following the LGPD means that those who use the platform can feel more trust and credibility. As a way to prevent identification and protect sensitive patient data, all identifying data is anonymized and identifier codes are used to reference them within the functionalities. To control the sharing of patient information, viewing, and editing are restricted only to physicians who belong to the same healthcare facility as the physician who entered the patient into the system. Physicians are eligible to participate in the registry after approval by their university's ethics committee.

In addition, for inclusion in the survey, patients and physicians must agree to the terms of use and consent that are included during the steps of self-registration on the platform, registration of a new patient, and completion of the self-report. Before providing their data, each patient, from the registry and self-report, provides consent to participate in the registry by signing the Informed Consent Form (ICF). The ICF is a required document to ensure that patients have clear and accurate information about the purpose and use of their data, as well as the possible risks and benefits involved in collecting and processing this data, making sure that they can refuse participation in the study or withdraw their consent at any time.

For more reliable data collection, all forms in the National Registry have been developed and validated by health professionals who work in outpatient clinics and are experts in the care of patients with ALS. Professionals from several hospitals in Brazil were invited, who work in ALS associations or are members of BAN. These professionals contributed to the consensus and definition of the indispensable variables to compose the minimum data set to be collected in the platform. After several rounds and cycles for data refinement and definition, three forms were developed: (1) a registration form made by physicians, (2) a self-report form, and (3) a Patient follow-up form (described in Section 3.1.1). The minimum data set defined by the professionals in question is made up of variables referring to personal and clinical data and the evolution of ALS in the patient. All defined forms are available as Supplementary material.

The National Registry platform was launched in August 2021 in partnership with the Brazilian MoH and the Ministry of Science and Technology. After its launch, publicity actions^1^ were carried out to obtain the largest adhesion between neurologists and ALS patients. At the moment, the platform is available on a publicly accessible web page (available at: https://revelanos.lais.ufrn.br/), which can be accessed and used freely by anyone who meets the access permission levels. The registry's homepage provides information about the RevELA project, with details about the study, access to manuals on how to use the system for physicians and patients, a list of partner centers, and a map showing the distribution of patients already registered in the system by states in Brazil.

#### 3.1.1. Physician's module

**Figure 2:**
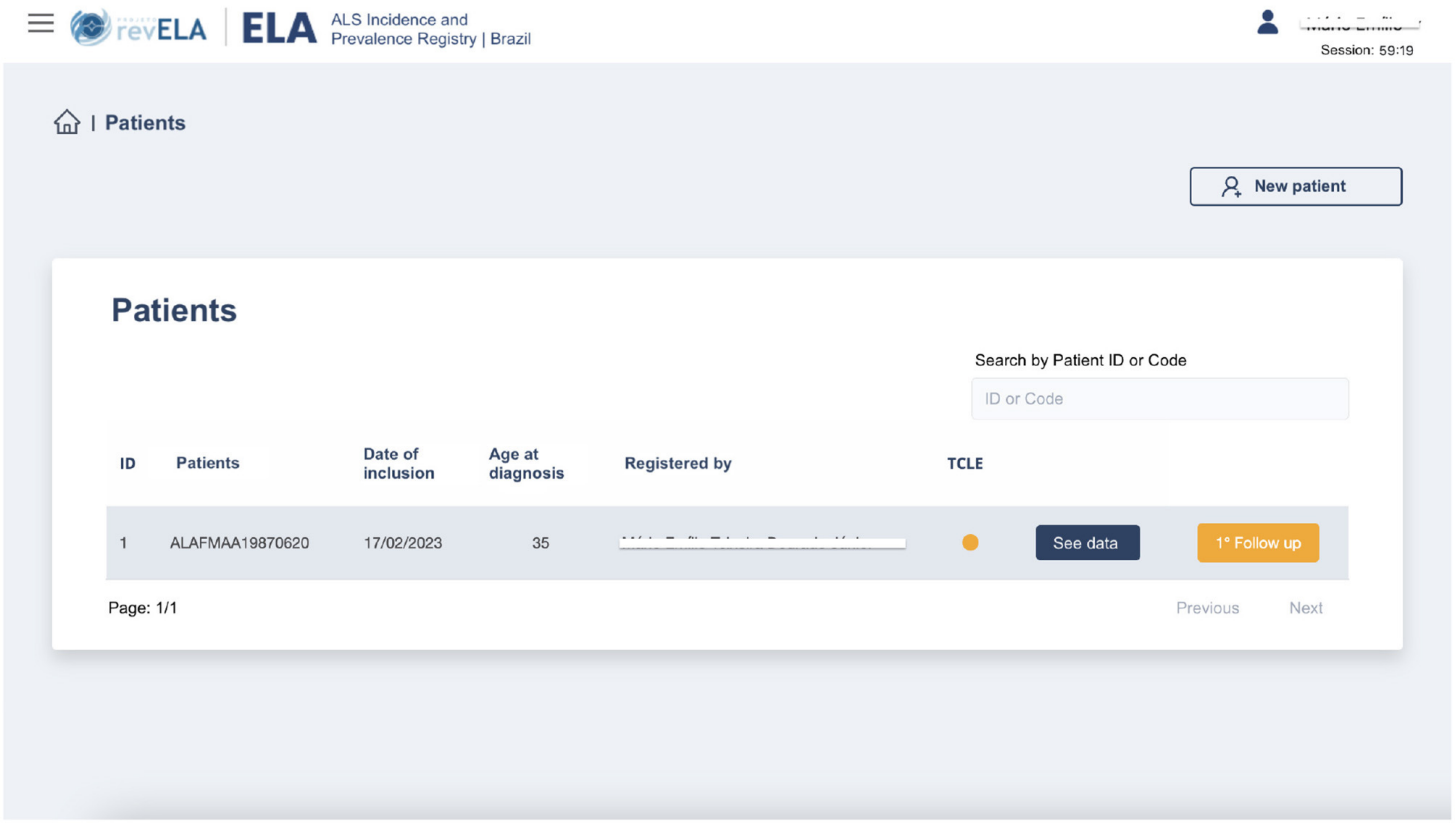
National Registry screen after physician user login. All data shown in this image are fictitious.

The neurologist module was developed to collect data from ALS patients through routine consultations with physicians in both public and private practice who have access to the National Registry platform. Thus, it is necessary that the physician, to add the registration of a new patient, be registered in the platform and be allowed to execute actions there. For this, the physician must self-register on the platform, adding their identification and login data. After this self-registration, a team managing the National Registry analyzes this data to validate the user's account. This registration validation step was implemented to prevent unauthorized users from having access to the actions and registration data. With the registration validated, the user can access the platform and its functionalities. Right after logging in, the user has access to the patient's list that has been added to the registry by the user (Figure 2), as well as other available functionalities, such as: viewing patient data, filling out the follow-up form, and registering a new patient.

**Figure 3:**
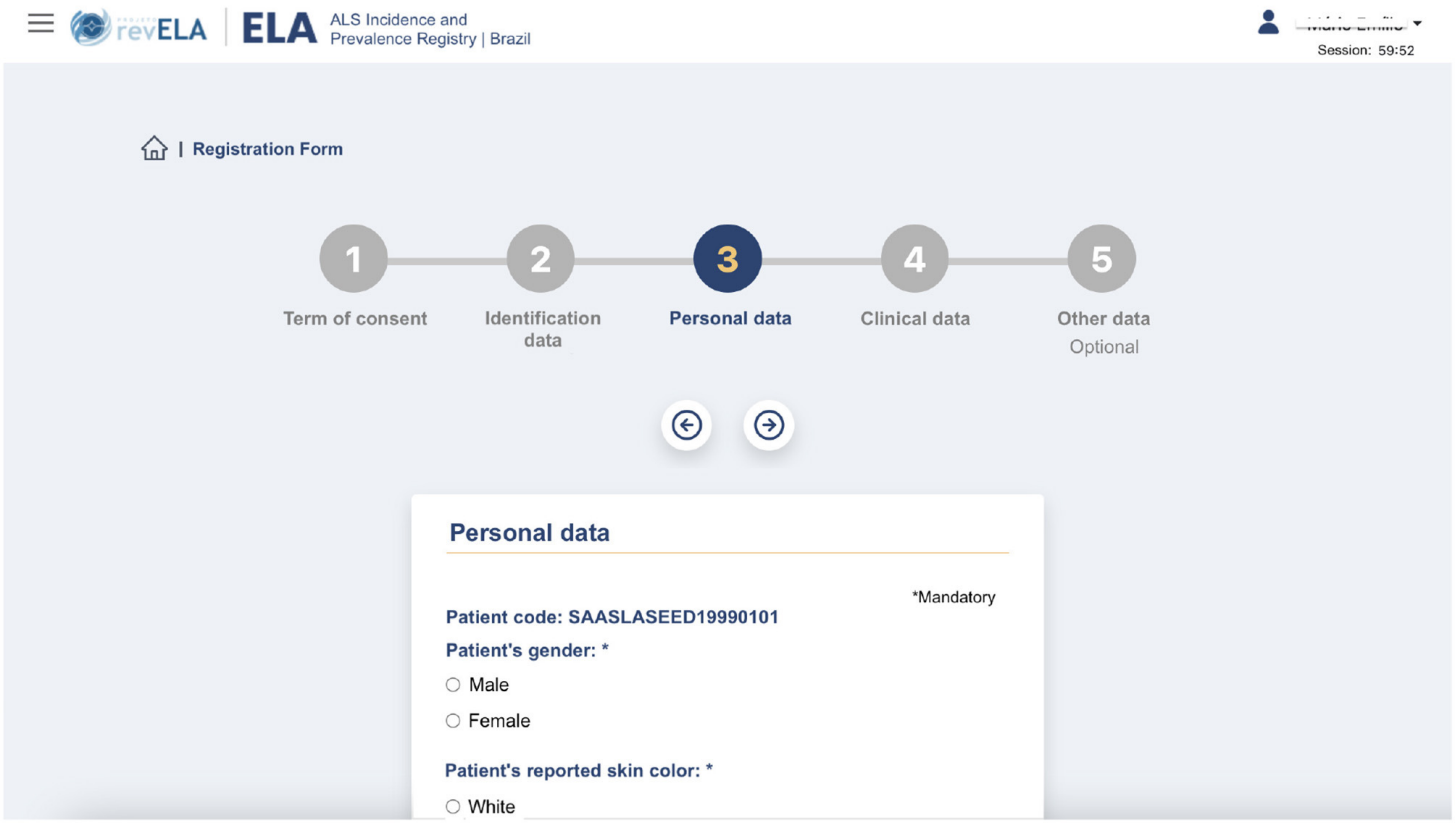
Registration screen of a new patient in the National Registry.

In the functionality of registering a new patient, the physician user fills out a series of questions containing personal, clinical, and disease progression follow-up data regarding the patient (Figure 3). Thinking of the user experience and avoiding screens with a large number of questions to be answered, this form was divided into five steps, as shown in Figure 3. Before introducing any data in the system, the patient must read and accept the ICF, signaling their agreement to participate and provide their data to the platform. Steps 2, 3, and 4 collect personal identification and clinical data, respectively. Step 5 is optional and collects data about the evolution of the disease. After filling in all the required data on the form (available in the Supplementary material), the physician saves the patient's data and enters it into the National Registry database.

When you add a patient to the platform, a unique identification key is generated with the patient's data. This key is used to prevent another physician from entering the same patient twice or more into the platform. Patient identification data is collected to avoid double registration. It is also worth noting that this data is stored in encrypted form and is protected by LGPD, which guarantees the security and anonymization of sensitive patient data.

**Figure 4:**
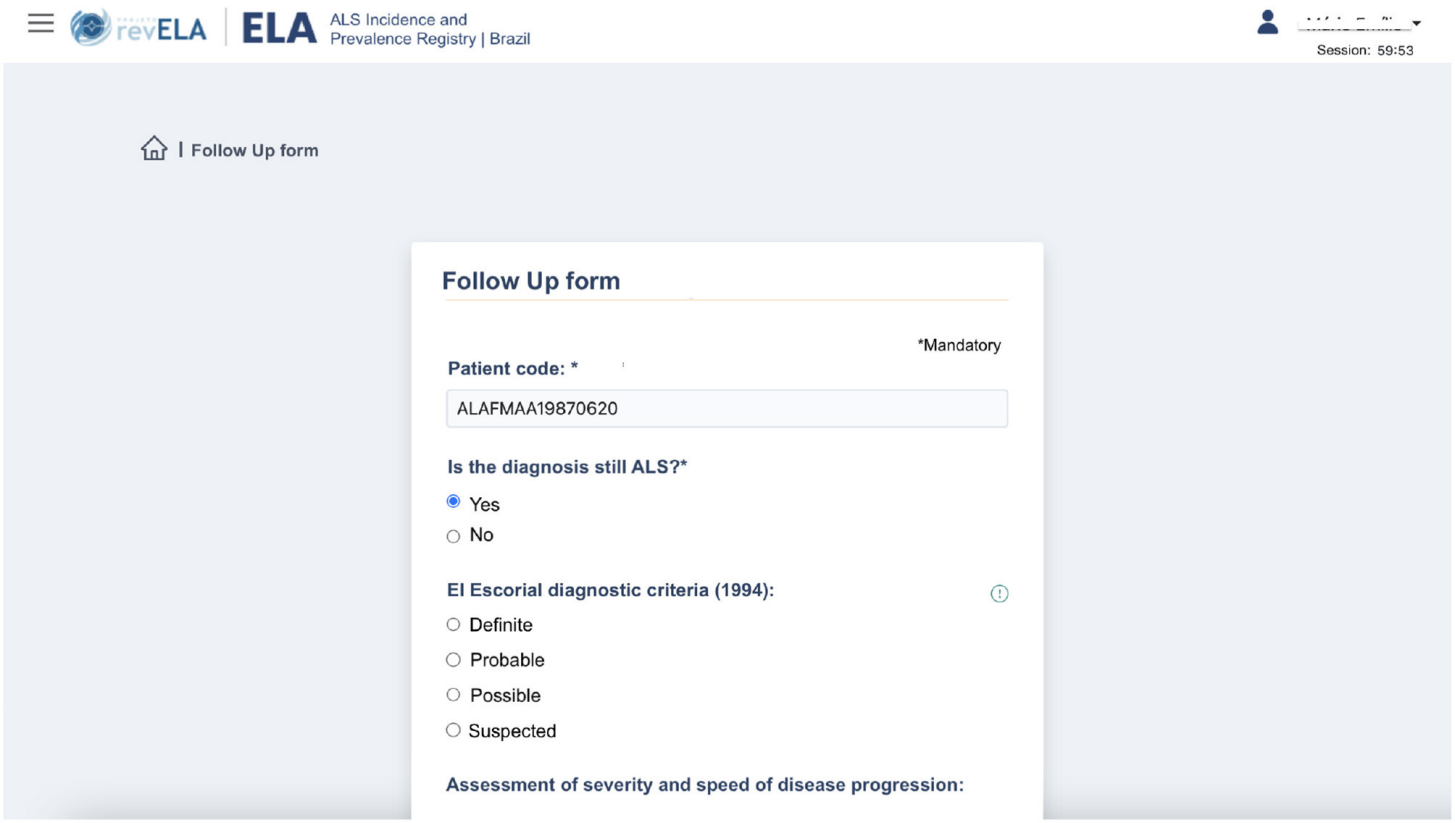
Follow up form screen for the ALS patients' monitoring.

Another important functionality available to the physician user is the completion of the follow-up form (Figure 4). This form is available for the physician to fill out every 3 months (a period defined by the specialists) on an optional basis, and its purpose is to record and evaluate the progression of the disease in each patient. These data are considered important to visualize the history of the disease in each patient. The main indicator for assessing disease progression is the Revised Amyotrophic Lateral Sclerosis Functional Rating Scale (ALSFRS-R), which is considered the gold standard for measuring ALS progression. Its criteria consist of a series of twelve physician-administered questions that assess basic functional activities such as eating, walking, dressing, and breathing (58).

#### 3.1.2. Patient module or self-report

The notification of ALS cases in Brazil is still characterized as non-mandatory. As a consequence, the concentration of information about the cases of patients with ALS in the country becomes even more complex and challenging. Thus, the self-report module developed in the National Registry emerges as a complementary strategy to reach the largest number of ALS patients' records in the whole Brazilian territory.

**Figure 5:**
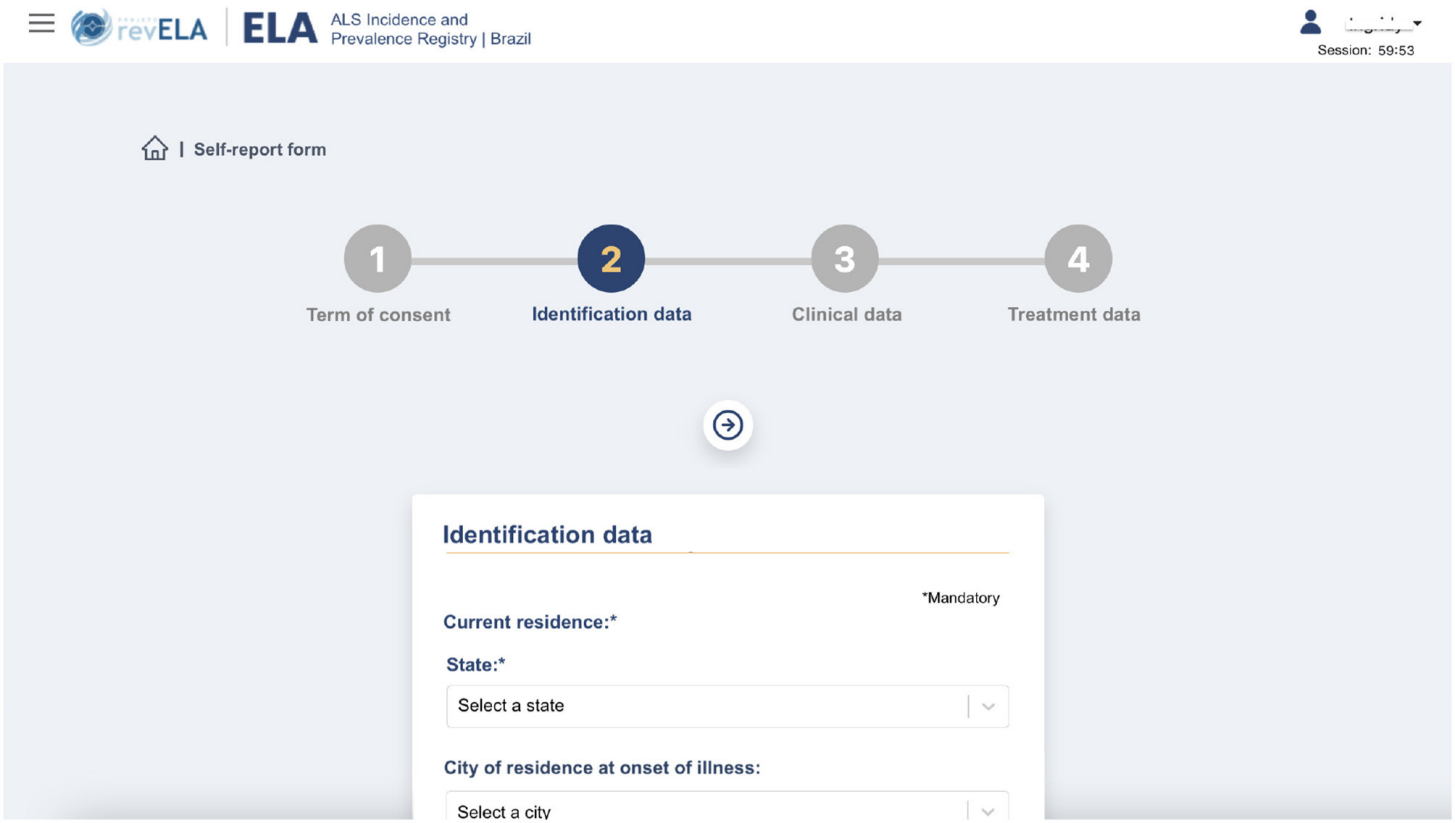
Self-report registration screen performed by the patient in the registry's platform.

This module was developed to allow the patient to contribute to the survey and add their data to the platform. Firstly, the patient or responsible caregiver performs self-registration, generating a user with access to the system. After verifying the informed email, the user can access the platform to add their data. The self-report form was divided into four steps. First, the patient accepts the ICF, a document indicating that the patient agrees to participate in the research and provide their data. Then, the patient fills out a series of forms containing identification (step 2), clinical (step 3), and treatment (step 4) data, similar to the registration done in the physician's module (Figure 5). Among the information requested to be filled in by the patient, the field for inserting the report is one of the main attributes for proving the ALS diagnosis during the self-report validation. It is through the analysis of this report that the physician team will conclude that the patient has indeed been diagnosed with ALS and the information will be included in the registry.

**Figure 6:**
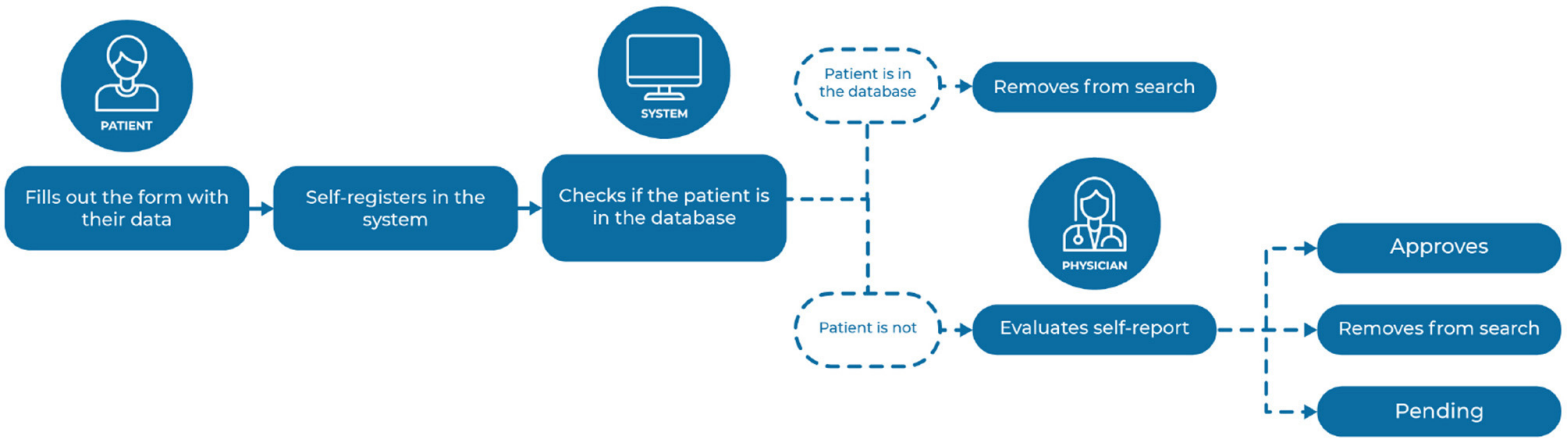
Patient data validation flow in the self-report.

All information provided by the patient goes through a validation flow so that a committee of physicians registered in the registry is responsible for analyzing the self-reports and adding them to the platform. This strategy was adopted to avoid the inclusion of data from people who were not diagnosed with ALS, generating underreporting. Regarding this validation process, shown in Figure 6, the physician on the committee can either approve the patient's self-report, indicating the data to be added into the database, or discard it, which means that the information will not be incorporated into the database on the platform. In addition, the physician can leave the patient self-report in 'Pending' status, representing an incomplete or incorrect data form. In this case, you can contact the patient and ask them to correct this data before finalizing the validation. Within the self-report approval functionality, it is allowed to add a comment to each one of them, justifying the withdrawal of the research, or even to leave some observation that may be visualized by other committee physicians.

When creating a patient user account for self-reporting on the platform, a unique identification key is also generated with the patient's data. This key is to prevent duplicate patients from both self-reporting and also from being recorded by physicians. Before adding the self-report to the database for the committee's validation, the system uses algorithms to check, by identifying data, if the patient exists in the registry database. If the algorithm finds repeating data, the patient's self-report is not added further to the database. This check is also performed in cases where the patient first performs the self-report and then is registered by the physician in the registry and in situations where another physician performs the patient registration for someone already on the system. Thus, the registry prevents duplicated data collection, contributing to a more accurate and complete analysis of ALS cases in Brazil.

### 3.2. Brazilian National ALS Observatory

To establish a specific and adequate environment for data visualization, a monitoring room was developed with epidemiological indicators generated from the data collected in the National Registry, called the Brazilian National ALS Observatory. This monitoring room is a complementary platform to the National Registry and is aimed essentially at presenting dashboards to help researchers and health managers visualize and analyze the data. Besides this, the observatory serves as a transparency portal for understanding the study results beyond the access to researchers and public managers.

The Brazilian National ALS Observatory arose from the need to display the data collected by the National Registry in an intuitive format that could improve the overview of case distribution and characteristics in the country. Grouping and cross-referencing between the different variables collected by the log and self-report stimulate discussions and comparisons of the analyses. Currently, access to this platform is limited to researchers and public health managers. The perspective is that in the future this access will become open to the entire population. It is noteworthy that the observatory is a read-only platform for patient data, and that it is not possible to add, remove or modify existing data in the National Registry database. Also, no personal or identifying data of the registered patients is displayed or reported.

**Figure 7:**
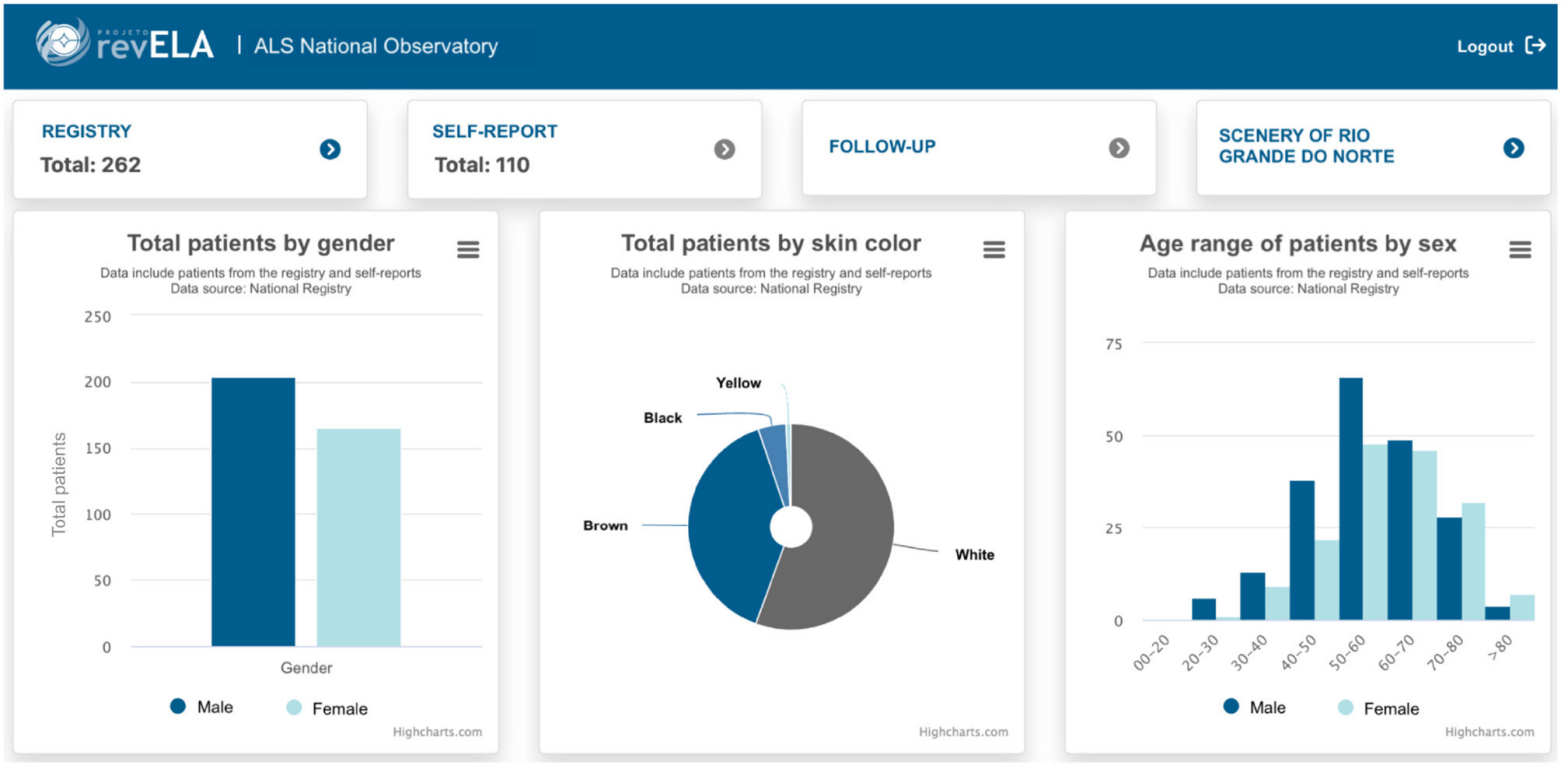
Home screen with general data collected through the National Registry.

The platform's home screen presents an overview of the data, displaying the number of logs made by physicians who have access to the registry or logs made through self-report, patient distribution by sex, age, and color (Figure 7). On this screen, you can also find tabs leading to other dashboards with more details, such as: a monitoring room with data only from the records made by the physician, with data only from self-report, with data only from the follow-up form, and the situation room per Brazilian state. Needless to say, the observatory was also developed and validated in collaboration with health professionals specializing in ALS, epidemiologists, statisticians, and data scientists.

**Figure 8:**
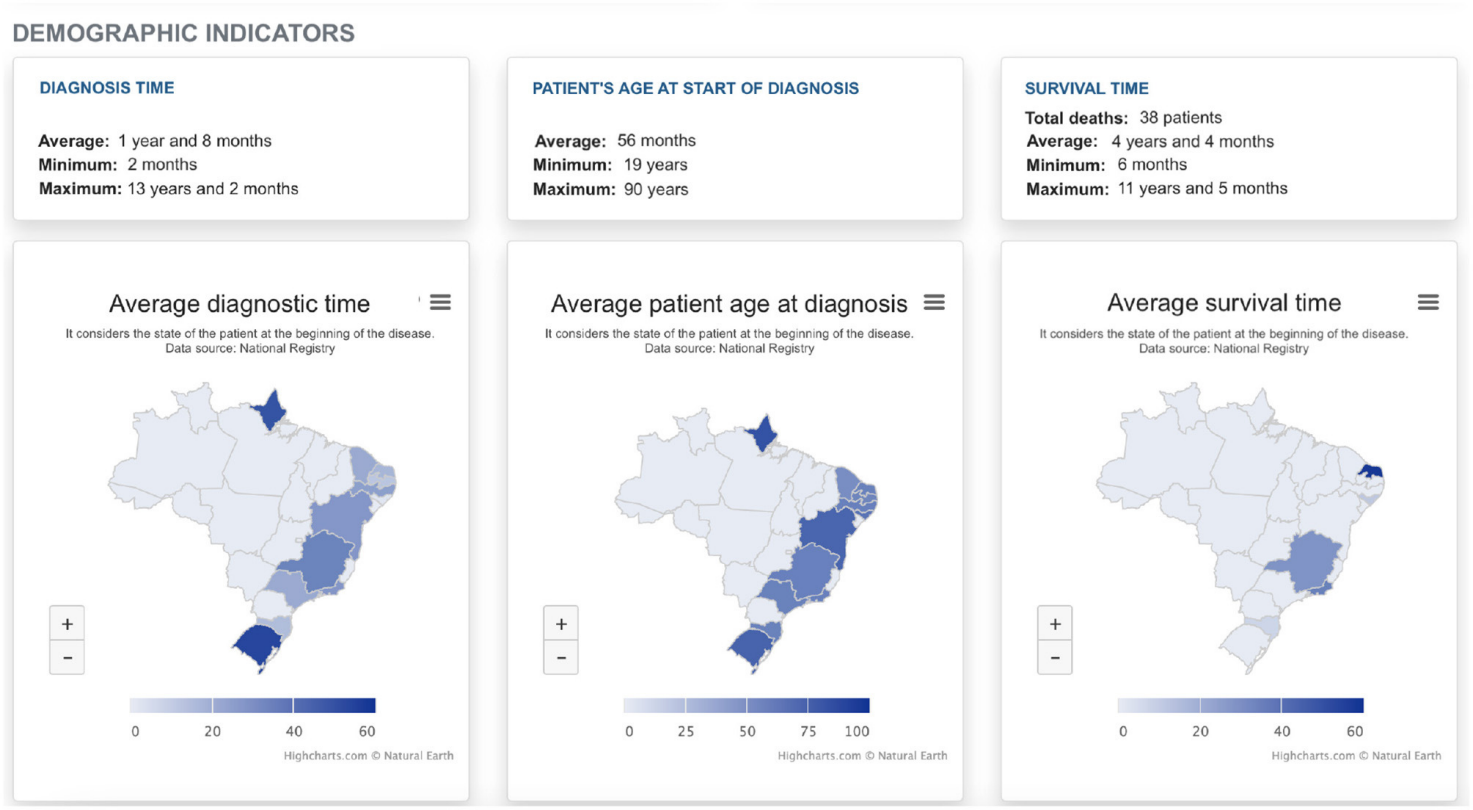
ALS indicators in Brazil.

Figure 8 presents ALS indicators in Brazil. This screen shows the minimum, maximum and average values of three different variables for patients from all over Brazil. Furthermore, some maps showing the average of these indicators for each state in the country are available. Providing these visualizations is fundamental to encourage discussions such as: understanding why the time to diagnosis may be discrepant among the states, comparing the average survival of patients in each state with the national average, and identifying the existence of patients with an early age of diagnosis, for example. These are just some of the analyses that can be performed with the graphics' assistance, demonstrating the importance of these representations, the impact that can be generated by them in the analyses, and the emergence of new insights.

In Brazil, the indicators related to patients diagnosed with ALS that were duly registered in the platform and whose data are presented by the National Observatory still do not portray the real country's epidemiological scenario. It bears mentioning that the ALS National Registry project is in the process of being consolidated in Brazil. This process occurs spontaneously throughout the country since the Brazil's MoH has not yet made the notifications and patients records on the platform compulsory, since the National ALS Registry is still in its 4th phase of research. It is worth mentioning that a law project has been processed and approved in the Brazilian Federal Senate which makes the notification of rare diseases, among them ALS, compulsory (59). This law project, after being approved in the Senate, was sent to the Brazilian Federal Chamber. Such a movement is essential so that all patients with ALS have their data compulsorily registered. In this context, the National ALS Registry is already adequate to absorb all the registries demanded by the Brazilian Health System.

**Figure 9:**
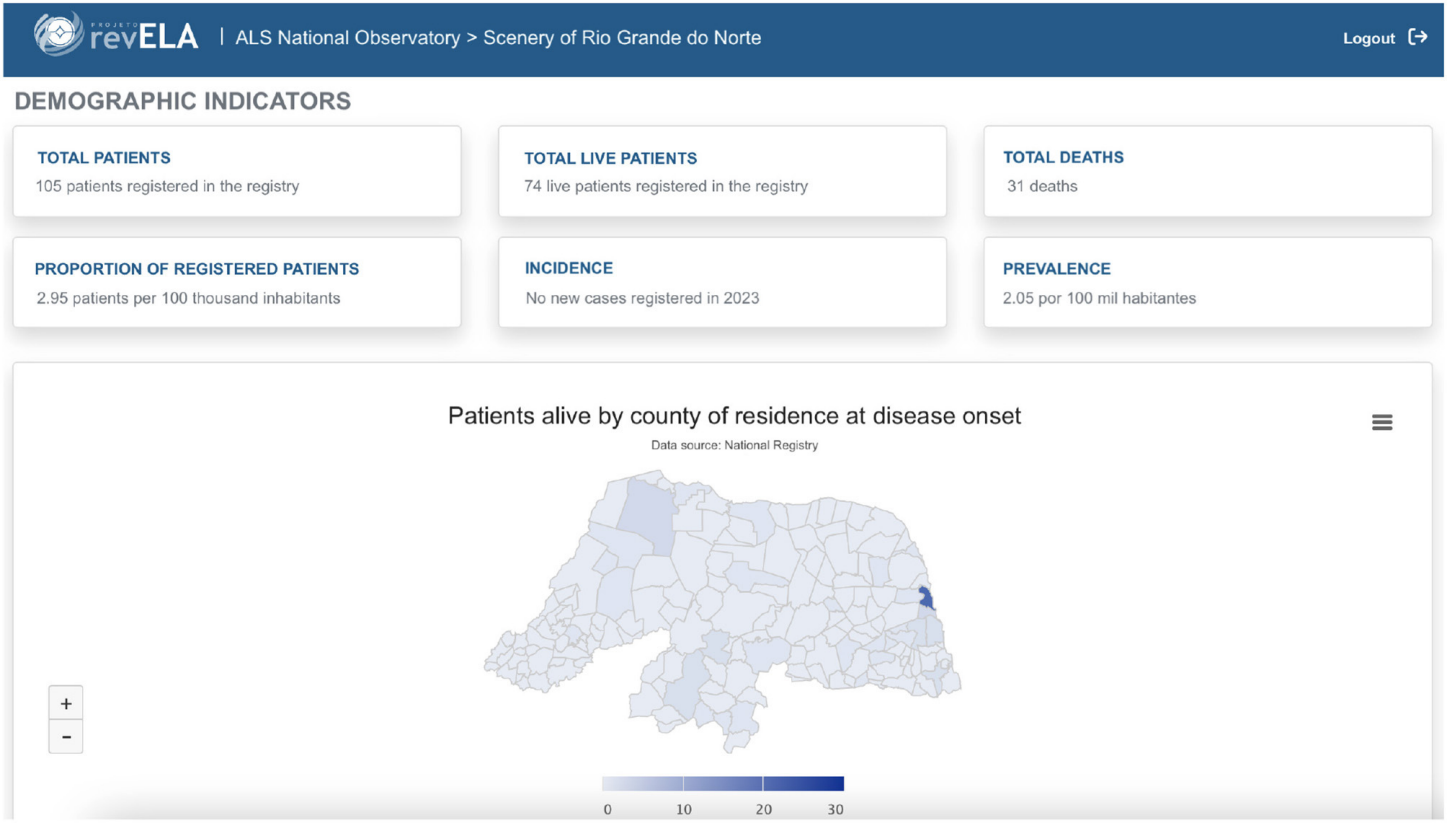
Indicators of ALS patients in the state of Rio Grande do Norte.

The ALS reference center located in the state of Rio Grande do Norte, where the digital solution was developed, joined the National Registry as a disease notification platform and carried out the patients' registration which is followed by the team. Figure 9 presents data on patients with ALS in the state of Rio Grande do Norte. Given this joining, the observatory emerges as a support tool for knowledge and decision-making related to ALS in the state. With the analysis made from the graphics available in the observatory, the public managers of the state had the initiative to develop public policies to improve care and promote more adequate care lines that provide life quality to patients with ALS.

### 3.3. Digital health solution architecture—National Registry and ALS Observatory

**Figure 10:**
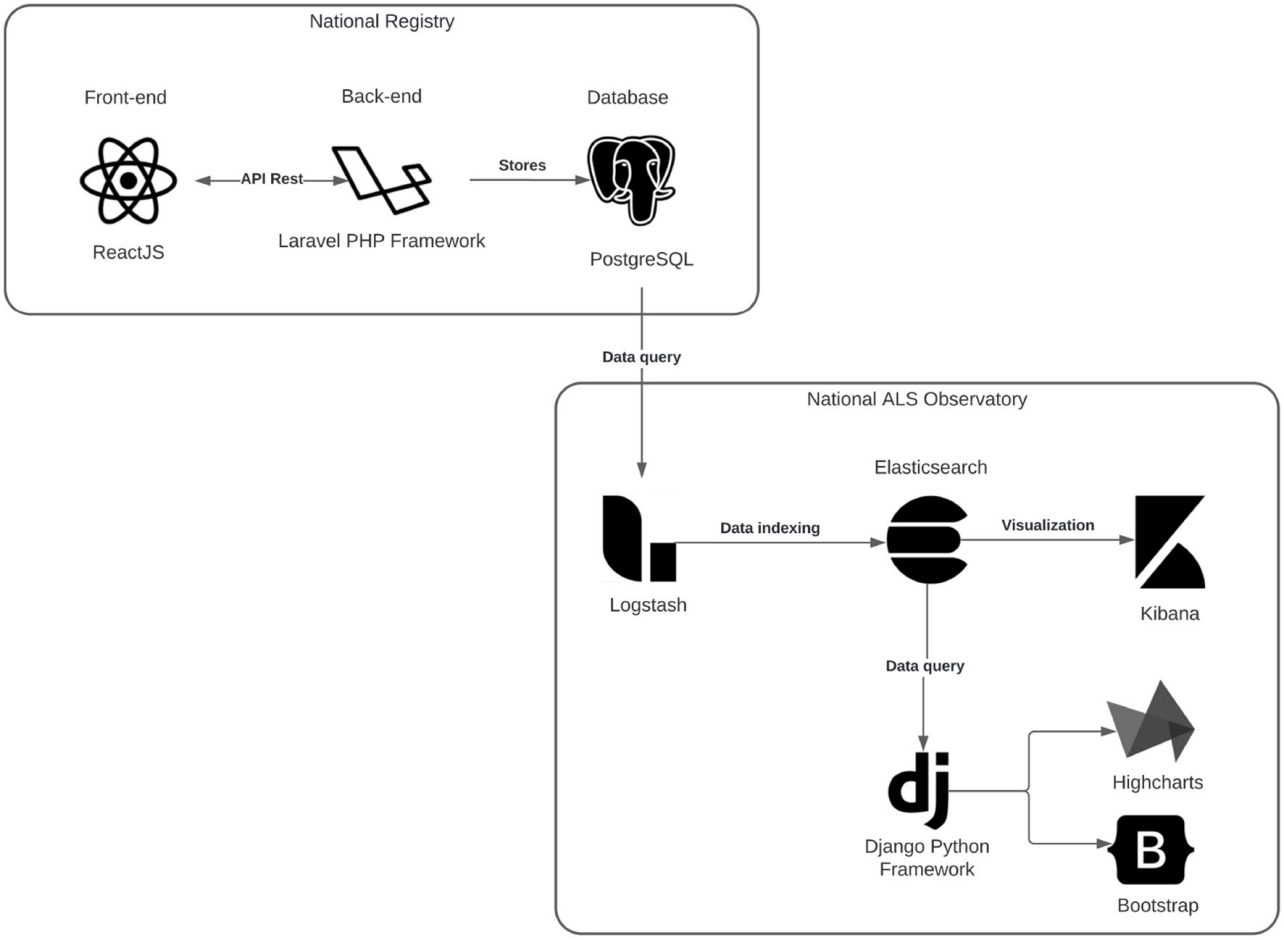
National Registry Architecture and ALS Observatory.

The proposed digital health solution are composed of two platforms with distinct but complementary objectives. For its development, several technology and tools were used, which are presented in Figure 10. The backend of the National Registry makes use of the Laravel PHP framework for the implementation of a REST API, which is a set of rules that are defined for applications and services to connect and communicate with each other. The frontend application connects to this REST API and renders the data through the ReactJS library for building user interfaces. Data persistence is done with PostgreSQL, a powerful relational database management system (DBMS).

The National ALS Observatory was also divided into two applications. The first application was created by designing a more efficient data usage facilitating the data manipulation and graph creation, for this the ELK Stack acronym for Elasticsearch, Logstash, and Kibana was used. Elasticsearch is a search and analysis engine, widely used and already well-validated not only in the academic field but also in the industry. Logstash is responsible for the loading process of the data collected from PostgreSQL and transferring it to Elasticsearch. Whereas Kibana allows you to visualize the data through graphs and charts in Elasticsearch. The second application concerns the web system and was developed using the Python Django framework for rendering the pages and graphics the Bootstrap and Highcharts libraries were used, respectively.

## 4. Discussion

ALS is still a neglected disease in the global health context, yet countries such as the USA (18, 20), Canada (19), Italy (21), Sweden (22) and New Zealand (23) have already implemented population-based registries for neuromuscular disease data collection. With this, these countries can visualize more accurately their epidemiological situation concerning this disease.

By supporting the development of RevELA, through Brazil's MoH, Brazil has contributed to an important advance and is now one of the few countries in the world that has the technology for reporting ALS. The next steps for Brazil should be to strengthen the National Registry of patients with ALS, focusing on the promotion of epidemiological studies that explore the entire extent of the disease, to overcome occasional studies, which are conducted only in some cities, regions, or states of the country. These studies, although important, are limited, especially when it comes to public health policies of national scope because they cannot provide adequate information for the country's public health authorities to develop more effective interventions at the national level.

Even with all the technological advances in health care in the world, ALS is still little known by science and health professionals. In the field of public health management, it will be possible to make more qualified decisions and adopt specific and more effective public policies, so that access to assistance, and distribution of medicines, and treatments can be expanded with more equity. These factors are following the guidelines of the Brazilian National Policy on Rare Diseases within the Brazilian Health System, Ordinance No. 199, of January 30, 2014 (60). This Ordinance of the Brazilian MoH represents a breakthrough in the care of patients with Rare Diseases, among them Amyotrophic Lateral Sclerosis. In addition, established the National Policy for the Comprehensive Care of People with Rare Diseases, and also approved the Guidelines for the Comprehensive Care of People with Rare Diseases within the Brazilian National Health System, and defined the financial incentives for funding. However, it was not possible to evaluate or measure the effectiveness of this policy due to the lack of qualified data and information about patients with Rare Diseases in Brazil. These aspects can be solved by joining the Brazilian National Registry of ALS Patients to collect data on the disease.

Influenced by the creation of the Brazilian National Registry of ALS patients, the state of Rio Grande do Norte (RN) in Brazil's Northeast Region is the first in the country to create and pass a compulsory notification law for ALS. Therefore, Law No. 10,924 of June 10, 2021 (61) purpose, is to register the cases of ALS in RN, compulsorily, and to evaluate some characteristics of the disease in the state. This aspect is very positive because it shows that other Brazilian states can follow the same path.

**Figure 11:**
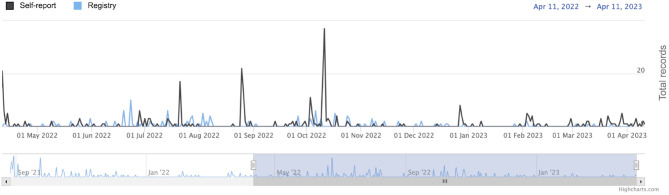
Timeline with the daily numbers of registrations and self-reports made in the National Registry.

In other Brazilian states, the ALS philanthropic associations became partners of the project and carry out campaigns to publicize the National Registry on social networks or in print media. As a result, the platform has gained more visibility in the states and there has been an increase in the number of patients who have self-reported. Figure 11 shows the timeline related to new registrations and self-reports performed in a given period. The evident peaks in the timeline are intrinsically associated with campaign periods and association disclosure events. Such campaigns are important strategies to minimize the limitation related to reaching the target population and to raise public awareness and access data about the disease. Thus, the more publicity there is, the more data will be collected in the country.

Brazil is a developing country and one of the largest economies in the world, but it is also a country with a lot of social inequality. By developing and incorporating digital health solutions into its healthcare system to increase the transparency and quality of information about patients with ALS, the country works toward providing access to healthcare services to lower-income patients who may develop ALS. In this case, the use of these technologies which also contribute to the digital transformation of health in the country, contribute not only improving the epidemiological view of this disease but also as a tool inducing equity and social justice. The qualification of the information about ALS in Brazil allows decision-makers to make public policies more effective, thereby increasing the impact of the health system on the quality of life of patients with ALS.

As a further perspective, the RevELA project has proposals to include other government sources for the data collection on ALS, promoting interoperability of health information systems, and contributing to a more accurate database that presents the reality of Brazil regarding ALS cases. In addition, due to the need for multidisciplinary follow-up, an Electronic Health Record for ALS Patients (EHR ALS) is being developed. This medical record consists of a platform for the electronic recording of data regarding the clinical history of patients with ALS intending to provide greater integration between the professionals that make up the multidisciplinary team and improve patient follow-up. To enable a detailed follow-up of patients' evolution, the National Registry will be integrated with the EHR ALS, thus creating a technological ecosystem centered on the ALS patient.

Just as it is being used for ALS, the digital health solution presented can serve as a basis for studies on other rare diseases in the country, seeking to understand more about their characteristics and the geographical distribution of patients. The formation of a registry with data on rare disease patients is the first step toward inducing public policies that will provide improvements in the quality of life of these individuals.

## 5. Conclusion

To minimize the problems concerning the absence of data on ALS in the country, the RevELA project, in partnership with the Brazilian MoH, proposed and developed a digital health solution that aims to efficiently collect and analyze clinical and epidemiological data of patients with ALS throughout the Brazilian territory. The data collected by the National Registry will be used in supporting essential surveillance and care actions for patients with ALS. This solution represents a key element in public policy strength and stands out for being the only ALS database, besides containing innovations that allow data collection by the health professional and/or by the patient. By using the data collected it is possible to investigate common characteristics among individuals, estimate the incidence and prevalence of cases, and find demographic characteristics and possible risk factors associated with the environment. To optimize the visualization of the collected data, a monitoring room was also developed consisting of ALS indicators in Brazil from data collected by the National Registry platform. It is believed that by using both platforms, it will be possible to understand the geographical distribution of cases, improve the care of individuals diagnosed with the disease, have the necessary knowledge to plan health interventions and support decision-making processes in the public health management dimension.

## Data availability statement

The original contributions presented in the study are included in the article/Supplementary material, further inquiries can be directed to the corresponding author.

## Author contributions

IB, AF, FF, MD, and RV contributed to the conception, study design, and development of the platform. TL, AM, and RL contributed to the design of the results and discussions. JH, PG, DN, JS, and JP contributed to the revision and writing of the manuscript. All authors contributed to the writing of the paper and approved the submitted version.

## Footnotes

^1^Brazilian National ALS Registry disclosure events 1° Encontro RevELA Brasil (http://igvb.org/blog/igvb-e-laboratorio-de-inovacao-tecnologica-da-ufrn-promovem-o-1o-encontro-revela-brasil/) Launch of the self-report platform (https://www.youtube.com/watch?v=nXGNkXEaak4).
