# Supplementary material for: Digital health solution for monitoring and surveillance of Amyotrophic Lateral Sclerosis in Brazil
Source: Front Public Health. 2023 Aug 25;11:1209633. doi: 10.3389/fpubh.2023.1209633 (PMC10485256; doi:10.3389/fpubh.2023.1209633)
Supplement: Supplementary file 1 [file Data_Sheet_1.PDF]

Supplementary Material

Template Document for Information from Data Collected on National ALS Registry Forms

This document presents Tables 1, 2 and 3 that describe the elements that comprise the Minimum Data Set (MDC) defined by experts. This MDC is part of the information model of the National Registry of Patients with ALS. The document has the following structure:

- **Section/Item**: description of the element or data.
- **Data Type**: demonstrates how to represent the element.
- **Value set**: indicates the value set that can be used to collect the item.
- **Notes**: conceptualizes or clarifies how to use the element.

Abbreviations:

ALS	Amyotrophic Lateral Sclerosis
IBGE	Instituto Brasileiro de Geografia e Estatística
ISO	International Organization for Standardization
SUS	Sistema Unico de Saúde
UF	Federative Unit

Table 1 - MDC from the National Register made by the Neurologist Physician

Section/Item	Data Type	Value set	Notes
**Identification data**			
Patient's Name	Text	-	-
Mother's Name	Text	-	-
Birthday Date	Date	ISO 8601 format	-
Phone Number	Text	-	Patient contact
State of residence	Encoded text	IBGE database - UFs	Patient's current state of residence
Municipality of residence	Encoded text	IBGE database - Cities	City of current residence of the patient
**Personal data**			
Gender	Encoded text	Female; Male	Patient's biological gender
Skin Color	Encoded text	White<br>Brown<br>Black<br>Yellow<br>Indigenous	Patient self-reported skin color
State of birth	Encoded text	IBGE database - UFs	Federative Unit (UF) where the patient was born
City of birth	Encoded text	IBGE database - Cities	City where the patient was born
Date of inclusion in the study	Date	ISO 8601 format	Date of patient data collection
**Clinical Data**			

Supplementary Material

Date of first symptom onset	Date	ISO 8601 format	-
Date of diagnosis	Date	ISO 8601 format	-
State of residence at onset of illness	Encoded text	IBGE database - UFs	Federative Unit (FU) where the patient lived at the beginning of the disease
City of residence at onset of illness	Encoded text	IBGE database - Cities	City where the patient lived at the beginning of the illness
Length of residence in the municipality	Numerical	-	Period that the patient lived in the municipality (in years and months)
Comorbidity	Encoded text	Yes<br>No	Patient's comorbidities
Family History of ALS	Encoded text	Yes<br>No	If there is a family member diagnosed with ALS
Family History of Dementia	Encoded text	Yes<br>No	If there is a family member with dementia
Location of first symptom onset	Encoded text	Right proximal upper limb (arm elevation)<br>Left proximal upper limb (arm elevation)<br>Right distal upper limb (hand weakness)<br>Left distal upper limb (hand weakness)<br>Right lower limb (leg)<br>Left lower limb (leg)<br>Shortness of breathe<br>Difficulty swallowing and gagging<br>Difficulty articulating words and phonation<br>unable to determine	Part of the body that manifested the first symptom
Motor Phenotypes	Encoded text	Classic ALS<br>Flail-arm syndrome (Vulpian-Bernhardt disease)<br>Flail-leg syndrome (pseudopolineuritic form)<br>Progressive Muscular Atrophy<br>Bulbar<br>Upper Motor Neuron Predominance Variant<br>Primary Lateral Sclerosis<br>Respiratory variant	Patient's motor phenotypes
Cognitive Status	Encoded text	Normal<br>behavioral dysfunction (behavioral variant)<br>Executive dysfunction (cognitive variant)<br>Behavioral and executive dysfunction<br>Frontotemporal Dementia<br>Not determined or not defined	Patient's current cognitive ability

El Escorial diagnostic criterion	Encoded text	Defined<br>Probable<br>Possible<br>Suspected	Adapted from Brooks BR et al: El Escorial World Federation of Neurology criteria for the diagnosis of amyotrophic lateral sclerosis. J Neurol Sci. 1994; Jul; 124(Suppl.):96-107 (63).
**Follow-up data**			
ALS functional scale	Numerical	0 to 48	Existing criteria in the ALS Functional Rating Scale - Revised
Riluzole	Encoded text	Yes<br>No	If the patient uses Riluzole.
Edaravone	Encoded text	Yes<br>No	If the patient uses Edaravone.
Non-invasive ventilation	Encoded text	Yes<br>No	If the patient uses non-invasive ventilation.
Tracheostomy	Encoded text	Yes<br>No	If the patient uses a tracheostomy.
Gastrostomy	Encoded text	Yes<br>No	If the patient uses a gastrostomy.
Death	Encoded text	Yes<br>No	If the patient died
Date of death	Date	ISO 8601 format	Date of death

Table 2 - MDC National Registration - Follow up

Section/Item	Data Type	Value set	Notes
**Demographic and clinical data**			
El Escorial diagnostic criterion	Encoded text	Defined<br>Probable<br>Possible<br>Suspected	Adapted from Brooks BR et al: El Escorial World Federation of Neurology criteria for the diagnosis of amyotrophic lateral sclerosis. J Neurol Sci. 1994; Jul; 124(Suppl.):96-107 (63).
ALS functional scale	Numerical	0 to 48	Existing criteria in the ALS Functional Rating Scale - Revised
Cognitive Status	Encoded text	Normal<br>behavioral dysfunction (behavioral variant)<br>Executive dysfunction (cognitive variant)<br>Behavioral and executive dysfunction<br>Frontotemporal Dementia<br>Not determined or not defined	Patient's current cognitive ability
Depression	Encoded text	Yes<br>No	If the patient has been diagnosed or has symptoms of depression.
Anxiety	Encoded text	Yes<br>No	If the patient shows characteristics of anxiety.
Pain	Encoded text	Yes<br>No	If the patient reports pain.
Riluzole	Encoded text	Yes<br>No	If the patient uses Riluzole.
Endaravone	Encoded text	Yes<br>No	If the patient uses Edaravone.

Bipap	Encoded text	Yes<br>No	If the patient uses non-invasive ventilation (bipap).
Tracheostomy	Encoded text	Yes<br>No	If the patient uses a tracheostomy.
Gastrostomy	Encoded text	Yes<br>No	If the patient uses a gastrostomy.
Tube feeding	Encoded text	Yes<br>No	If the patient uses a feeding tube.
AMBU Exercise	Encoded text	Yes<br>No	If the patient used or uses AMBU to exercise.
CoughAssist	Encoded text	Yes<br>No	If the patient has used or is using CoughAssist.
Communication Technology	Encoded text	Yes<br>No	If the patient made or makes use of communication technology
Multidisciplinarity offered	Encoded text	Yes<br>No	If the patient has follow-up with other health professionals.
Home care	Encoded text	Yes<br>No	If the patient has home care.
Death	Encoded text	Yes<br>No	If the patient died.

Tabela 3 - MDC - National Registration - Self-report

Section/Item	Data Type	Value set	Notes
**Identification data**			
Patient Initials	Text	-	-
Mother's Initials	Text	-	-
Birthday Date	Date	ISO 8601 format	-
Phone Number	Text	-	Patient contact
State of residence	Encoded text	IBGE database - UFs	Patient's current state of residence
Municipality of residence	Encoded text	IBGE database - Cities	City of current residence of the patient
Gender	Encoded text	Female; Male	Patient's biological gender
Skin Color	Encoded text	White<br>Brown<br>Black<br>Yellow<br>Indigenous	Patient self-reported skin color
Some health professional has told you that you possibly have Amyotrophic Lateral Sclerosis (ALS)	Encoded text	Yes<br>No	If the patient is suspected of having ALS
You were clinically diagnosed with ALS	Encoded text	Yes<br>No	If the patient has ever been diagnosed with ALS
You were evaluated by a neurologist	Encoded text	Yes<br>No	If the patient has already been evaluated by a neurologist
**Clinical Data**			
Date of first symptom onset	Date	ISO 8601 format	-
Date of diagnosis	Date	ISO 8601 format	-

State of residence at onset of illness	Encoded text	IBGE database - UFs	Federative Unit (FU) where the patient lived at the beginning of the disease
City of residence at onset of illness	Encoded text	IBGE database - Cities	City where the patient lived at the beginning of the illness
Length of residence in this municipality	Numerical	-	Period that the patient lived in this municipality (in years and months)
State of birth	Encoded text	IBGE database - UFs	Federative Unit (FU) where the patient was born
City of birth	Encoded text	IBGE database - Cities	City where the patient was born
Family History of ALS	Encoded text	Yes<br>No	If there is a family member diagnosed with ALS
Location of first symptom onset	Encoded text	Right proximal upper limb (arm elevation)<br>Left proximal upper limb (arm elevation)<br>Right distal upper limb (hand weakness)<br>Left distal upper limb (hand weakness)<br>Right lower limb (leg)<br>Left lower limb (leg)<br>Shortness of breathe<br>Difficulty swallowing and gagging<br>Difficulty articulating words and phonation<br>unable to determine	Location of the body that manifested the first symptom
**Treatment dataIdentification data**			
Riluzole	Encoded text	Yes<br>No	If the patient uses Riluzole.
Endaravone	Encoded text	Yes<br>No	If the patient uses Edaravone.
Bipap	Encoded text	Yes<br>No	If the patient uses non-invasive ventilation (bipap).
Tracheostomy	Encoded text	Yes<br>No	If the patient uses a tracheostomy.
Gastrostomy	Encoded text	Yes<br>No	If the patient uses a gastrostomy.
Tube feeding	Encoded text	Yes<br>No	If the patient uses a feeding tube.
AMBU Exercise	Encoded text	Yes<br>No	If the patient used or uses AMBU to exercise.
CoughAssist	Encoded text	Yes<br>No	If the patient has used or is using CoughAssist.
Communication Technology	Encoded text	Yes<br>No	If the patient made or makes use of communication technology.
Multidisciplinarity offered	Encoded text	Yes<br>No	If the patient has follow-up with other health professionals.
Home care	Encoded text	Yes<br>No	If the patient has home care.
Did or do alternative treatments	Encoded text	Yes<br>No	-
Patient Care Type	Encoded text	Public health service – SUS<br>Health insurance plan	-

		Private professionals	
